# TROPHY registry — status report

**DOI:** 10.1007/s00381-021-05258-w

**Published:** 2021-06-29

**Authors:** U. W. Thomale, C. Auer, P. Spennato, A. Schaumann, P. Behrens, S. Gorelyshev, E. Bogoslovskaia, A. Shulaev, A. Kabanian, A. Seliverstov, A. Alexeev, O. Ozgural, G. Kahilogullari, M. Schuhmann, R. Jimenez-Guerra, N. Wittayanakorn, A. Sukharev, J. Marquez-Rivas, S. Linsler, A. El Damaty, P. Vacek, M. Lovha, R. Guzman, S. Stricker, T. Beez, C. Wiegand, M. Azab, D. Buis, M. Sáez, S. Fleck, C. Dziugan, A. Ferreira, T. Radovnicky, C. Bührer, S. Lam, S. Sgouros, J. Roth, S. Constantini, S. Cavalheiro, G. Cinalli, A. V. Kulkarni, H. C. Bock

**Affiliations:** 1 Pediatric Neurosurgery, Campus Virchow Klinikum, Charité Universitätsmedizin, Berlin Augustenburger Platz 1, 13353 Berlin, Germany; 2 Division of Pediatric Neurosurgery, Kepler Universitätsklinikum, Linz, Austria; 3 Pediatric Neurosurgery, AORN Santobono Pausilipon, Naples, Italy; 4 Pediatric Neurosurgery, Moscow Bashlyaeva Pediatric Hospital, Moscow, Russia; 5 Pediatric Neurosurgery, Surgut Clinical Perinatal Center, Surgut, Russia; 6 Pediatric Neurosurgery, Children’s Republic Clinical Hospital, Kazan, Russia; 7 Pediatric Neurosurgery, Children’s Regional Hospital, Krasnodar, Russia; 8 Pediatric Neurosurgery, Kemerovo Regional Pediatric Hospital, Kemerovo, Russia; 9 Pediatric Neurosurgery, Chelyabinsk Regional Children’s Clinical Hospital, Chelyabinsk, Russia; 10 Neurosurgery, Ankara University, Ankara, Turkey; 11 Pediatric Neurosurgery, University Hospital of Tübingen, Tubingen, Germany; 12 Neonatal Neurosurgery, National Institute of Perinatology, Mexico City, Mexico; 13 Surgery, Queen Sirikit National Institute of Child Health, Bangkok, Thailand; 14 Pediatric Neurosurgery, Regional Children Hospital, Yekaterinburg, Russia; 15 Neurosurgery, Virgen del Rocio Hospital, Seville, Spain; 16 Neurosurgery, Saarland University Hospital, Homburg, Saarland Germany; 17 Pediatric Neurosurgery, Heidelberg University Hospital, Heidelberg, Germany; 18 Neurosurgery, University Hospital and Faculty of Medicine in Pilsen, Charles University, Pilsen, Czech Republic; 19 Neurosurgery, Volyn Regional Pediatric Hospital, Lutsk, Ukraine; 20 Neurosurgery, Universitätskinderspital Beider Basel, Basel, Switzerland; 21 Neurosurgery, Heinrich-Heine-University, Duesseldorf, Germany; 22 Neurosurgery, Marienhospital, Osnabrück, Germany; 23 Neurosurgery, Damietta Specialized Hospital, Damietta, Egypt; 24 Neurosurgery, Amsterdam University Medical Centres, Amsterdam, Netherlands; 25 Neurosurgery, Hospital La Paz, Madrid, Spain; 26 Neurosurgery, University Medicine Greifswald, Greifswald, Germany; 27 Pediatric Neurosurgery, Ann & Robert H. Lurie Children’s Hospital of Chicago, Chicago, USA; 28 Neurosurgery, Centro Hospitalar Universitário São João, Porto, Portugal; 29 Neurosurgery, Masaryk Hospital, Usti Nad Labem, Czech Republic; 30 Pediatric Neurosurgery, Iaso Childrens Hospital, Athens, Greece; 31 Pediatric Neurosurgery, Tel Aviv Medical Center, Tel Aviv, Israel; 32 Pediatric Neurosurgery, Federal University of Sao Paulo, Sao Paulo, Brazil; 33 Pediatric Neurosurgery, Sick Children Hospital, University of Toronto, Toronto, Canada; 34 Pediatric Neurosurgery, University Medical Center Göttingen, Gottingen, Germany

**Keywords:** Infant hydrocephalus, Posthemorrhagic hydrocephalus, TROPHY, External ventricular drainage, Ventricular access device, Subgaleal shunt, Neuroendoscopic shunt

## Abstract

**Introduction:**

The TROPHY registry has been established to conduct an international multicenter prospective data collection on the surgical management of neonatal intraventricular hemorrhage (IVH)-related hydrocephalus to possibly contribute to future guidelines. The registry allows comparing the techniques established to treat hydrocephalus, such as external ventricular drainage (EVD), ventricular access device (VAD), ventricular subgaleal shunt (VSGS), and neuroendoscopic lavage (NEL). This first status report of the registry presents the results of the standard of care survey of participating centers assessed upon online registration.

**Methods:**

On the standard of treatment forms, each center indicated the institutional protocol of interventions performed for neonatal post-hemorrhagic hydrocephalus (nPHH) for a time period of 2 years (Y1 and Y2) before starting the active participation in the registry. In addition, the amount of patients enrolled so far and allocated to a treatment approach are reported.

**Results:**

According to the standard of treatment forms completed by 56 registered centers, fewer EVDs (Y1 55% Y2 46%) were used while more centers have implemented NEL (Y1 39%; Y2 52%) to treat nPHH. VAD (Y1 66%; Y2 66%) and VSGS (Y1 42%; Y2 41%) were used at a consistent rate during the 2 years. The majority of the centers used at least two different techniques to treat nPHH (43%), while 27% used only one technique, 21% used three, and 7% used even four different techniques. Patient data of 110 infants treated surgically between 9/2018 and 2/2021 (13% EVD, 15% VAD, 30% VSGS, and 43% NEL) were contributed by 29 centers.

**Conclusions:**

Our results emphasize the varying strategies used for the treatment of nPHH. The international TROPHY registry has entered into a phase of growing patient recruitment. Further evaluation will be performed and published according to the registry protocol.

## Introduction

Neonatal IVH with consecutive hydrocephalus is a complex disease in respect to the fragile newborn mostly premature babies, which is not rarely accompanied with other relevant comorbidities. Previous surveys as well as the recent guidelines of the treatment of pediatric hydrocephalus have shown that no consistent treatment recommendations currently exist for the treatment of neonatal posthemorrhagic hydrocephalus (nPHH) [1–3]. The major aims of the neurosurgical treatment are to reduce secondary brain damage by stabilizing the hydrocephalus and avoiding treatment-related complications. Future perspective must be directed towards establishing a better standardized guideline [4].

The long-term goal of the TROPHY registry is to contribute substantial data to further develop treatment guidelines for neonates with posthemorrhagic hydrocephalus [5]. This status report presents the data resulting from the standard of treatment forms to show the distribution of surgical techniques used at the participating centers before active participation as well as the current status of data collection indicated by the number of centers and patients included so far in the TROPHY registry.

## Methods

The TROPHY registry was introduced to facilitate a prospective comparison of the most established methods of the neurosurgical treatment of nPHH in an international multicenter context. Through an online-based process of data collection and evaluation, external ventricular drainage (EVD), ventricular access device (VAD), ventricular subgaleal shunt (VSGS), and neuroendoscopic lavage (NEL) will be compared at the participating centers, with respect to perioperative complications, mortality, shunt dependency, amount of shunt revisions, ventricular width at 6, 12, and 24 months and neurological outcome at 24, 36, and 60 months [5].

### Infrastructure

A password-protected access to the online registry application is provided for the participating centers via https://trophy-registry.org. FileMaker® Server software is used for the central data server hosted at the Charité Universitätsmedizin (Berlin, Germany). Data privacy is guaranteed by SSL encryption. Once included in the registry, patients’ data are pseudonymized via an automatically generated case ID and recorded in electronic case report forms (eCRF).

During the first step of online registration, each center has to complete an eligibility form stating their standard treatment for nPHH during the two previous years before active participation can be initiated. These data include the following:

- total amount of neurosurgical interventions performed in neonates
- total amount of neuroendoscopic interventions performed in neonates
- total amount of neurosurgical interventions in nPHH according to VAD, EVD, VSGS, NEL, or other specific techniques
- shunt rate for nPHH from individual experience

### Status

For evaluating the current status of the registry, the number of patients entered from 9/2018 to 2/2021 in the database was evaluated, including the initial interventions performed for nPHH.

### Statistics

All values are given as median and range. Possible difference between the numbers of interventions per year was assessed by Wilcoxon matched paired test. The proportion of centers using a specific intervention per year was evaluated by chi-square test. For graphical illustration, the software Prism 8 (GraphPad, USA) or Excel (Microsoft, USA) was utilized.

## Results

**Fig. 1:**
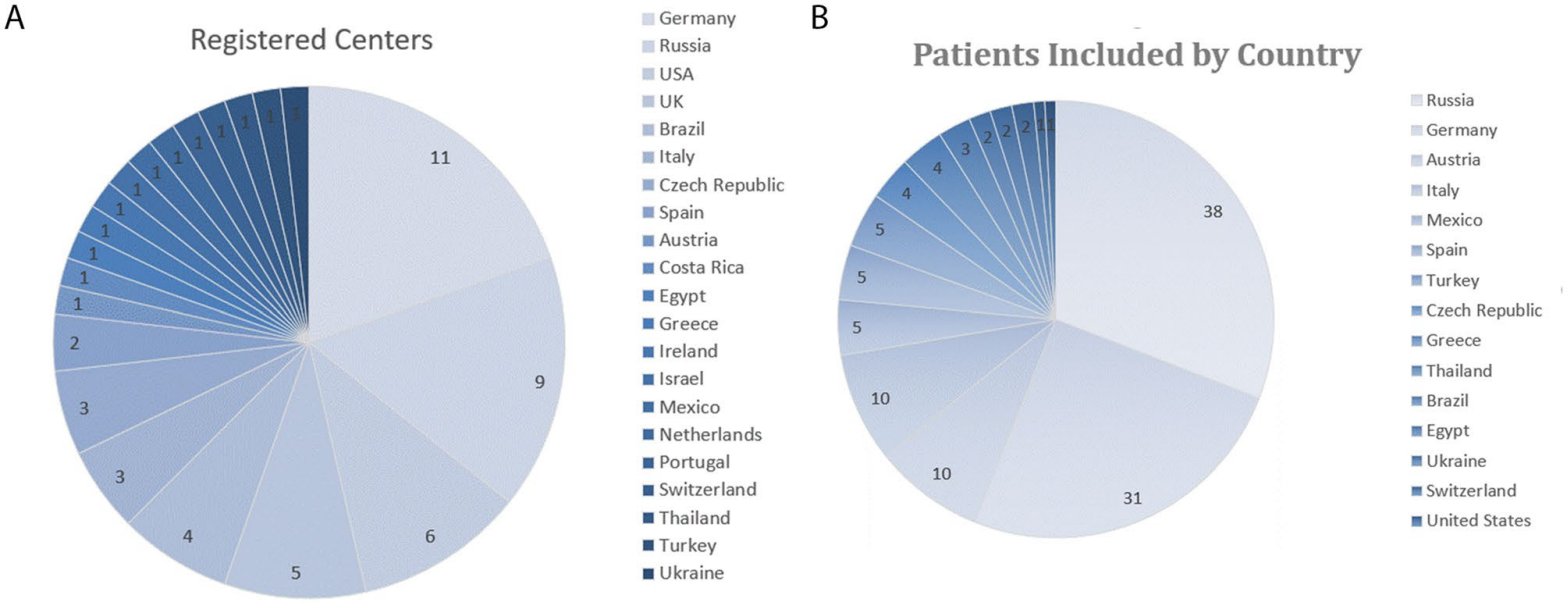
International participation in the TROPHY registry. **A** Distribution of centers per country registered. **B** Amount of cases enrolled per country

From September 2018 to February 2021, a total of 56 centers completed an eligibility form stating the standard treatment each center used before active participation in TROPHY. Twenty-nine centers have been actively contributing cases. In terms of international distribution for registration, the three most active countries are Germany, Russia, and the USA, while the most actively contributing countries are Russia, Germany, and Italy as well as Austria (Fig. 1A, B).

For these 56 centers, the number of annual interventions per center according to the standard treatment forms was 35 ± 42 neurosurgical interventions for both years and 9 ± 14 neuroendoscopic interventions in year 1 (Y1) and 11 ± 16 in year 2 (Y2).

**Fig. 2:**
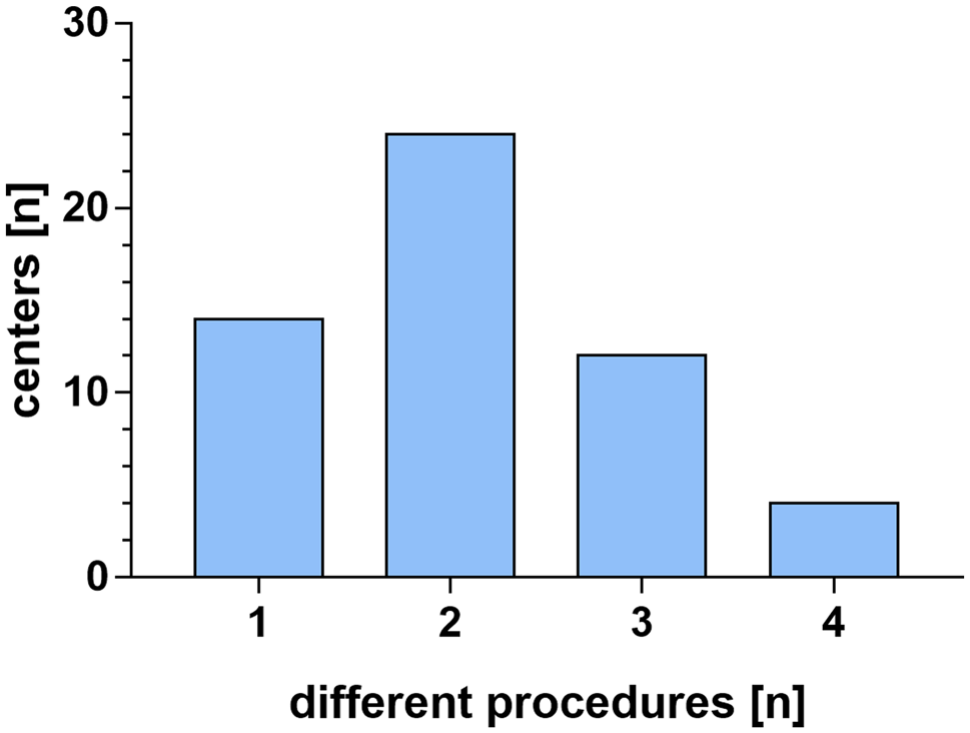
Number of different interventions used per center according to the standard treatment forms

An overview of the different treatment options for nPHH is given in Table 1. Compared to the initially utilized interventions, there was a non-significant decrease in the percentage of centers that used EVD from year 1 to year 2 (55% vs. 46%) with a median number of interventions per center of 4 (1–34) in Y1 and 4.5 (1–32) in Y2. VAD (Y1 66%; Y2 66%) and VSGS (Y1 43%; Y2 41%) were used in a similar percentage of centers in both years with a similar median number of interventions per center (VAD: Y1: 5(1–29); Y2: 4(1–32); VSGS: Y1: 7(1–32); Y2: 6(1–49)). For NEL, there was a non-significant increase in the percentage of centers using this technique (Y1 39%; Y2 52%; *p* = 0.055), and there was a significantly greater median number of intervention per center (Y1 3(1–15); Y2 3(1–17); *p* < 0.01). Other techniques were used in 13 (23%) centers with similar number of interventions per center each year (Y1 5(1–17); Y2 4(1–21)). Among those, 6 centers used initial shunting, 2 endoscopic ventriculocisternostomy (ETV), 2 ETV and choroid plexus coagulation (CPC), 1 drainage irrigation fibrinolytic therapy (DRIFT) protocol, 1 septostomy, and 1 without specification. The estimated shunt dependency rate of 54 centers exhibited a median of 75% (range 20–100%). In most of the centers, 2 different techniques for initial treatment of nPHH were used (*n* = 24). Only 14 centers were dedicated to one technique while 12 centers used 3 techniques and 4 centers used 4 different techniques for the treatment of nPHH (Fig. 2).

**Table 1:** Treatment regimen for nPHHC

Interventions in neonates	Centers providing technique n (%)		Interventions conducted per center median (range)	
	Year 1	Year 2	Year 1	Year 2
External ventricular drainage	31 (55)	26 (46)	4 (1–34)	4.5 (1–32)
Ventricular access device	37 (66)	37 (66)	5 (1–29)	4 (1–35)
Ventricular subgaleal shunt	24 (43)	23 (41)	7 (1–32)	6 (1–49)
Neuroendoscopic lavage	22 (39)	29 (52) **	3 (1–15)	3 (1–17)*
Other interventions^a^	13 (23)	13 (23)	5 (1–17)	4 (1–21)
Shunt dependency rate	*n* = 54		71% (20–100)	

^*^p < 0.05 versus Y1; ^**^p = 0.06 versus Y1

^a^Shunt: *n* = 6; endoscopic third ventriculocisternostomy and choroid plexus coagulation (ETV + CPC): *n* = 2; endoscopic third ventriculostomy (ETV): *n* = 2; drainage, irrigation, and fibrinolytic therapy (DRIFT): *n* = 1; septostomy: *n* = 1; not specified: *n* = 1

**Fig. 3:**
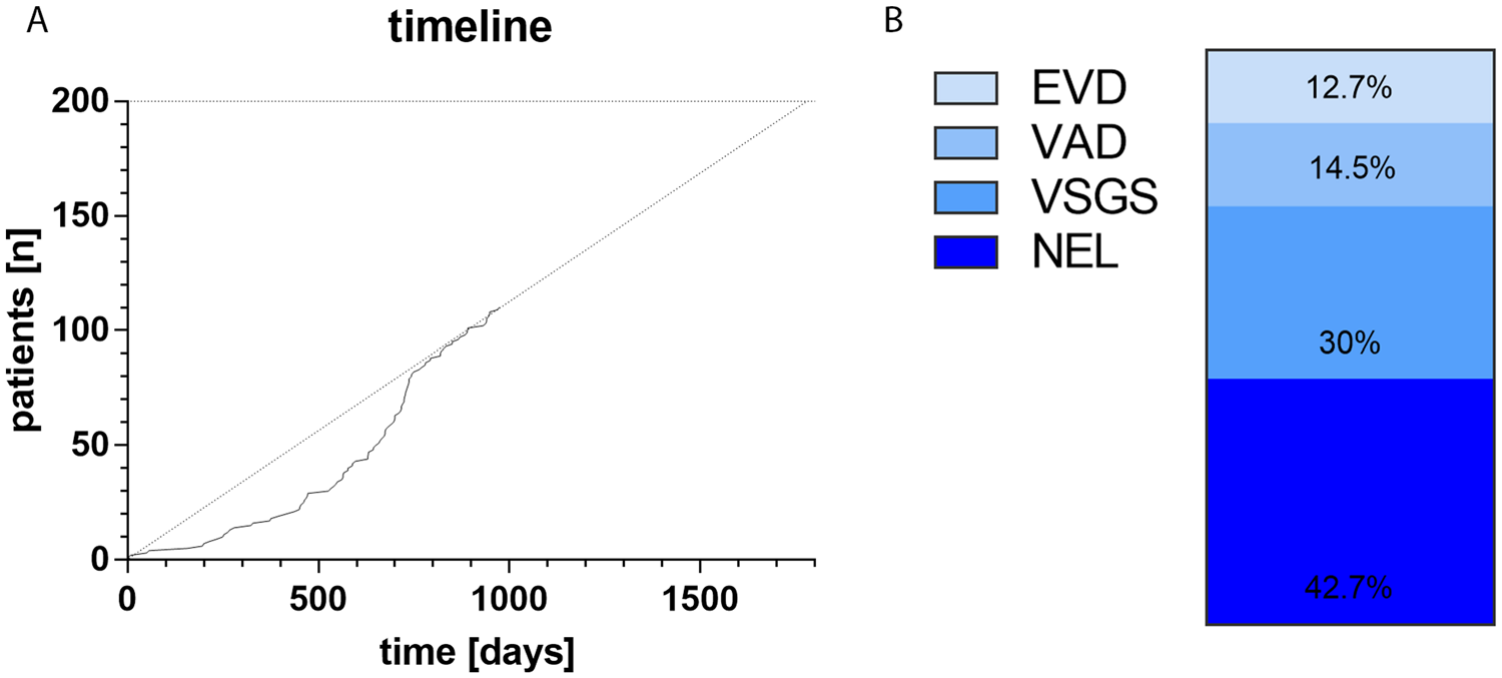
**A** Total number of patients enrolled in the TROPHY registry over time. **B** Techniques used as initial treatment for nPHHC in the enrolled patient cohort

During the active enrolment of patients into the TROPHY registry, 110 patients have so far been included over 973 days from 29 centers (Fig. 3A). The proportion of techniques used is NEL in 42.7%, VSGS in 30%, VAD in 14.5%, and EVD in 12.7% (Fig. 3B).

## Discussion

The first evaluation of the standard treatment forms filled-in by the centers upon registration for TROPHY as well as the activity for patient enrollment provides an overview of the current status of the registry. We are more than grateful for all the participating centers from all over the world. The initial interest in study participation is reflected by the high number of centers registered so far. Of these centers, a little more than half are actively contributing centers, which largely relates to the different administrative processes and local regulatory rules necessary before study participation can be started.

The results from the standard treatment form show the heterogeneity of different approaches among the centers as well as within the centers for the treatment of nPHH. The fact that only 14 centers were dedicated to one technique, and most were using two or more techniques reflects heterogeneous patients or heterogenous philosophies or both. In addition, we have seen that there is some dynamic change in the utilization of neuroendoscopic lavage, which was more often used in the second year before TROPHY participation compared to the first year. This might reflect promising results published recently on this technique [6–10]. For the other techniques, VAD and VSGS appeared to be used consistently across the 2 years, while EVD was used non-significantly less in the second year, which is in line with recent reviews about the topic [4, 11].

It is too early to speculate the degree to which, for example, very low birth infants are treated differently at registry centers compared to nearly term infants with IVH. It is also not clear if neonates with high amount of blood volume intraventricularly might need more aggressive interventions compared to low grade IVH neonates. Such questions remain in the individual decision making of the centers or the surgeons but will need further investigation in the future. Thus, the necessity of generating recommendations for a standardized guideline through an international registry study like TROPHY is emphasized.

The activity for TROPHY registry has shown to be decent so far. Further enthusiasm and support of activity are necessary in the future in order to keep the pace for sufficient data acquisition. The recently published data from the DRIFT cohort 10 years follow-up suggest that more aggressive evacuation of blood from the ventricles in the early phase of nPHH might lead to ongoing decrease in severe disability resulting from the initial burden of the disease [12, 13] and a decrease in health care costs for these chronically affected children. We hope that the TROPHY registry might also be able to answer questions like this for the widely used techniques for management of nPHH.

The shunt rate from the survey shows a median of 75% with quite a wide range. These numbers must be interpreted with caution since it may reflect an individual view rather than a careful retrospective evaluation of data. In the DRIFT long-term evaluation, the shunt rate was 39% in the DRIFT group and 33% in the control group, respectively [13]. It will be important to elucidate if shunt dependency at all is a relevant outcome parameter or if a reasonable neurocognitive development will be much more important to achieve with or without a shunt. Future investigations will have to compare their results to the DRIFT data to find out if any other technique of early treatment of nPHH will lead to similar results in long term. TROPHY will remain active to contribute to this challenge in the future.

## References

[1] Badhiwala JH, Hong CJ, Nassiri F, Hong BY, Riva-Cambrin J, Kulkarni AV (2015) Treatment of posthemorrhagic ventricular dilation in preterm infants: a systematic review and meta-analysis of outcomes and complications. J Neurosurg Pediatr 16(5):545–55. 10.3171/2015.3.PEDS14630 10.3171/2015.3.PEDS14630 26314206

[2] Christian EA, Melamed EF, Peck E, Krieger MD, McComb JG (2016) Surgical management of hydrocephalus secondary to intraventricular hemorrhage in the preterm infant. J Neurosurg Pediatr 17(3):278–84. 10.3171/2015.6.PEDS15132 10.3171/2015.6.PEDS15132 26565942

[3] Kumar N, Al-Faiadh W, Tailor J, Mallucci C, Chandler C, Bassi S et al (2017) Neonatal post-haemorrhagic hydrocephalus in the UK: a survey of current practice. Br J Neurosurg 31(3):307–11. 10.1080/02688697.2016.1226260 10.1080/02688697.2016.1226260 27687144

[4] Bauer DF, Baird LC, Klimo P, Mazzola CA, Nikas DC, Tamber MS et al (2020) Congress of neurological surgeons systematic review and evidence-based guidelines on the treatment of pediatric hydrocephalus: update of the 2014 guidelines. Neurosurgery 87(6):1071–5. 10.1093/neuros/nyaa434 10.1093/neuros/nyaa434 34791462

[5] Thomale UW, Cinalli G, Kulkarni AV, Al-Hakim S, Roth J, Schaumann A et al (2019) TROPHY registry study design: a prospective, international multicenter study for the surgical treatment of posthemorrhagic hydrocephalus in neonates. Childs Nerv Syst 35(4):613–9. 10.1007/s00381-019-04077-4 10.1007/s00381-019-04077-4 30726526

[6] Behrens P, Tietze A, Walch E, Bittigau P, Bührer C, Schulz M et al (2020) Neurodevelopmental outcome at 2 years after neuroendoscopic lavage in neonates with posthemorrhagic hydrocephalus. J Neurosurg Pediatr 1–9. 10.3171/2020.5.PEDS20211 10.3171/2020.5.PEDS20211 32764179

[7] d'Arcangues C, Schulz M, Bührer C, Thome U, Krause M, Thomale UW (2018) Extended experience with neuroendoscopic lavage for posthemorrhagic hydrocephalus in neonates. World Neurosurg 116:e217-e24. 10.1016/j.wneu.2018.04.169 10.1016/j.wneu.2018.04.169 29729459

[8] Etus V, Kahilogullari G, Karabagli H, Unlu A (2018) Early endoscopic ventricular irrigation for the treatment of neonatal posthemorrhagic hydrocephalus: a feasible treatment option or not? a multicenter study. Turk Neurosurg 28(1):137–41. 10.5137/1019-5149.JTN.18677-16.0 10.5137/1019-5149.JTN.18677-16.0 27759873

[9] Schulz M, Bührer C, Pohl-Schickinger A, Haberl H, Thomale UW (2014) Neuroendoscopic lavage for the treatment of intraventricular hemorrhage and hydrocephalus in neonates. J Neurosurg Pediatr 13(6):626–35. 10.3171/2014.2.PEDS13397 10.3171/2014.2.PEDS13397 24702621

[10] Tirado-Caballero J, Rivero-Garvia M, Arteaga-Romero F, Herreria-Franco J, Lozano-Gonzalez A, Marquez-Rivas J (2020) Neuroendoscopic lavage for the management of posthemorrhagic hydrocephalus in preterm infants: safety, effectivity, and lessons learned. J Neurosurg Pediatr:1–10. 10.3171/2020.2.PEDS2037 10.3171/2020.2.PEDS2037 32413865

[11] El-Dib M, Limbrick DD, Inder T, Whitelaw A, Kulkarni AV, Warf B et al (2020) Management of post-hemorrhagic ventricular dilatation in the infant born preterm. J Pediatr. 10.1016/j.jpeds.2020.07.079 10.1016/j.jpeds.2020.07.079 PMC8297821 32739263

[12] Luyt K, Jary S, Lea C, Young GJ, Odd D, Miller H et al (2019) Ten-year follow-up of a randomised trial of drainage, irrigation and fibrinolytic therapy (DRIFT) in infants with post-haemorrhagic ventricular dilatation. Health Technol Assess 23(4):1–116. 10.3310/hta23040 10.3310/hta23040 PMC6398084 30774069

[13] Luyt K, Jary SL, Lea CL, Young GJ, Odd DE, Miller HE et al (2020) Drainage, irrigation and fibrinolytic therapy (DRIFT) for posthaemorrhagic ventricular dilatation: 10-year follow-up of a randomised controlled trial. Arch Dis Child Fetal Neonatal Ed 105(5):466–73. 10.1136/archdischild-2019-318231 10.1136/archdischild-2019-318231 PMC7547901 32623370

